# Metabolic phenotypes in primary unknown metastatic carcinoma

**DOI:** 10.1186/1479-5876-12-2

**Published:** 2014-01-06

**Authors:** Hye Min Kim, Do Hee Kim, Woo Hee Jung, Ja Seung Koo

**Affiliations:** 1 Department of Pathology, Yonsei University College of Medicine, Severance Hospital, 50 Yonsei-ro, Seodaemun-gu, Seoul, South Korea; 2 Department of Pathology, Severance Hospital, Brain Korea 21 PLUS Project for Medical Science Yonsei University College of Medicine, Seoul South Korea

**Keywords:** Carcinoma, Primary unknown, Metabolism

## Abstract

**Background:**

The purpose of this study is to evaluate expression of metabolism-related proteins in primary unknown metastatic carcinoma (PUMC) and associated implications for treatment.

**Methods:**

A tissue microarray containing 77 cases of PUMC was constructed and immunohistochemical staining was used to evaluate expression of the following proteins: Glycolysis-related: Glut-1, carbonic anhydrase (CA) IX, and monocarboxylate transporter (MCT) 4; Glutaminolysis-related: glutaminase1 (GLS1), glutamate dehydrogenase (GDH), and amino acid transporter-2 (ASCT2); and Mitochondrial-related: ATP synthase, succinate dehydrogenase (SDH)A, and SDHB. The association between immunohistochemical staining results and clinicopathologic parameters was evaluated.

**Results:**

The expression of metabolism-related proteins was different depending on the histologic subtype. Compared to other subtypes, squamous cell carcinomas (SQ) expressed more Glut-1 (p = 0.028), while adenocarcinomas (AD) expressed more SDHB in the stroma (p = 0.025). The expression of metabolism-related proteins was also different depending on the clinical subtypes. Glut-1 was expressed most in the nodal type and the least in carcinomatosis type, when compared to other subtypes (p = 0.021). The metabolic phenotypes also showed other trends: when the stroma showed no glutaminolysis, the tumor mostly invaded lymph node, bone, and brain, while the tumor invaded regions other than lymph node, bone, and brain when the stroma showed glutaminolysis (p = 0.003). When the stroma showed the mitochondrial metabolic type, the histologic subtype was mainly AD, but the non-mitochondrial type was associated more with SQ (P = 0.049).

**Conclusion:**

For PUMC, the expression of metabolism-related proteins, such as Glut-1 and SDHB, differs in the tumor or stroma depending on the clinical and histologic tumor subtype.

## Background

Primary unknown metastatic carcinoma (PUMC) is defined as metastatic carcinoma with no definitive primary tumor identified from clinical symptoms, patient history, radiologic imaging, laboratory investigation, histologic evaluation, or immunohistochemical staining [1]. PUMC is a heterogeneous disease which accounts for 5 ~ 15% of malignant tumors [2-4]; histologically, it consists of adenocarcinomas (AD) (50 ~ 60%), poorly differentiated carcinomas (PD) (30 ~ 40%), and other histologic types, including squamous cell carcinomas (SQ) (5 ~ 8%) and undifferentiated carcinomas (UD) (2-5%) [4,5]. The exact nature of PUMC is not well defined, but one hypothesis is that it is a true metastatic tumor without an identifiable primary focus, while another hypothesis is that it is an unusual primary tumor which simulates metastatic disease [5].

In general, cancer cells show different metabolic characteristics from normal cells. Normal cells obtain energy by oxidative phosphorylation, while cancer cells use glycolysis. It is called Warburg effect theory [6]. Thus, glycolysis is one of the most important components of cancer metabolism. However, glycolysis, while a major characteristic of cancer cell metabolism, cannot account for energy usage in all types of cancer cells. According to the literature, the dominant metabolic process can be either glycolysis or oxidative phosphorylation based on the tumor type [7]. In addition to glucose metabolism, glutamine metabolism is also an important cancer cell metabolic pathway. Glutamine metabolism is important in that it both produces ATP and provides intermediates for macromolecular synthesis [8]. Therefore, glycolysis, glutaminolysis, and mitochondrial metabolism are likely to play indispensable roles in tumor metabolism.

There are studies about metabolism-related protein expression in various cancers. Most of the metabolism-related protein expression differ among clinicopathologic parameter of cancer [9-14], it can be suggested that PUMC also show different expression of metabolism-related protein expression, however there isn’t enough study.

The purpose of this study is to evaluate metabolism-related protein expression in PUMC and associated implications for treatment.

## Methods

### Patient selection and clinicopathologic evaluation

This study utilized formalin-fixed, paraffin-embedded (FFPE) tissue samples from patients ultimately diagnosed with PUMC at Severance Hospital. All patients were diagnosed with metastatic carcinoma by pathologists from January 1999 to December 2012. Cases with only a small amount of biopsy material were excluded. All archival hematoxylin and eosin (H&E)–stained slides were reviewed for each case. The clinicopathologic parameters evaluated in each tumor included patient age, sex, histological type, involved organ, and patient outcome. According to the histologic standards, PUMCs were categorized into four subtypes [5]: AD showed glandular differentiation within the tumor; SQ showed evidence of squamous differentiation, such as intercellular bridges and keratin pearls; PD did not show differentiation to any specific lineage; and UD were composed of syncytial tumor cell nests or singly scattered tumor cells closely intermingled with dense lymphoplasmacytic infiltration similar to nasopharyngeal undifferentiated carcinoma. Also, according to the clinical and radiologic diagnosis, PUMCs were categorized into subtypes: 1) “nodal type” if the tumor involved only the lymph node; 2) “single organ type” if the metastatic tumor involved only one organ (other than lymph nodes); for example, metastatic adenocarcinoma of brain or bone with unknown primary origin; 3) “intermediate type” in which two organs were involved; and 4) “carcinomatosis type” if three or more organs were involved. The study was approved by the Institutional Review Board of Severance Hospital (4-2012-0606).

### Tissue microarray (TMA)

Among the H&E–stained slides, the most appropriate FFPE tumor tissue samples were gathered retrospectively, the most representative tumor area was marked, the selected area was extracted with a punch machine, and the 3 mm tissue core was inserted into the 6×5 recipient block. The TMA was constructed with two tissue cores for all cases.

### Immunohistochemistry (IHC)

The antibodies used for IHC in this study are shown in Additional file 1: Table S1. Using xylene and alcohol solution, the 3 mm-thick slices from the FFPE tissue block were deparaffinized and rehydrated, and then a Ventana Discovery XT automated stainer (Ventana Medical Systems, Tucson, AZ, USA) was used. Antigen retrieval was performed with CC1 (Cell Conditioning 1) buffer (citrate buffer pH 6.0, Ventana Medical Systems). IHC was performed, including the appropriate positive and negative controls. The primary antibody incubation step was omitted in the negative control. For each antibody, the positive controls recommended by the manufacturers were used.

### Interpretation of immunohistochemical results

IHC result interpretation was based on the product of the proportion of stained cells and the immunhistochemical staining intensity. A product between 0–1 was called negative and a product between 2–6 was called positive [9]. The proportion of stained cells was scored as 0 for negative, 1 for positive with less than 30%, and 2 for positive with greater than or equal to than 30%. Immunhistochemical staining intensity was scored as 0 for negative, 1 for weak, 2 for moderate, and 3 for strong. For the interpretation of immunohistochemical TMA, the interpretation criteria above is applied to all two cores if there were any difference in the staining.

### Statistical analysis

Data were statistically processed using SPSS for Windows version 12.0 (SPSS Inc., Chicago, IL). Student’s *t* test and Fisher’s exact test were used for continuous and categorical variables, respectively. Statistical significance was assumed when p < 0.05. Kaplan-Meier survival curves and log-rank statistics were employed to evaluate time to survival. Multivariate regression analysis was performed using a Cox proportional hazards model.

## Results

### Basal characteristics of PUMC patients according to the histologic subtypes

The basal characteristics of 77 PUMC cases, based on their histologic subtypes, are summarized in Table 1. AD accounted for 27 (35.1%) cases, PD for 16 (20.8%) cases, SQ for 20 (26.0%) cases, and UD for 14 (18.2%) cases. Depending on the histologic subtypes, the clinical subtypes were also different. While AD consisted of more of the carcinomatosis subtype, PD, SQ, and UD consisted more of the nodal subtype (p < 0.001). Different histologic subtypes indicated different postoperative treatments: AD cases were treated mostly with chemotherapy, PD and SQ with chemo-radiation therapy, and UD with only surgery (p = 0.039).

**Table 1:** Clinicopathologic characteristics of patients

**Clinical parameters**	**Total N = 77 (%)**	**Histologic subtype**				**p-value**
		**AD (n = 27) (%)**	**PD (n = 16) (%)**	**SQ (n = 20) (%)**	**UD (n = 14) (%)**	
Age (years, mean ± SD)	55.4 ± 11.8	59.3 ± 12.3	55.3 ± 12.1	55.0 ± 9.6	48.3 ± 10.6	0.042
Sex						0.521
Female	29 (37.7)	13 (48.1)	6 (37.5)	6 (30.0)	4 (28.6)	
Male	48 (62.3)	14 (51.9)	10 (62.5)	14 (70.0)	10 (71.4)	
Clinical subtype						**<0.001**
Nodal type	34 (44.2)	3 (11.1)	8 (50.0)	14 (70.0)	9 (64.3)	
Single organ type	15 (19.5)	6 (22.2)	5 (31.3)	0 (0.0)	4 (28.6)	
Intermediate type	14 (18.2)	7 (25.9)	1 (6.3)	5 (25.0)	1 (7.1)	
Carcinomatosis type	14 (18.2)	11 (40.7)	2 (12.5)	1 (5.0)	0 (0.0)	
Organs involved						0.102
Lymph node	49 (63.6)	12 (44.4)	9 (56.3)	19 (95.0)	9 (64.3)	
Bone	10 (13.0)	6 (22.2)	2 (12.5)	0 (0.0)	2 (14.3)	
Brain	9 (11.7)	4 (14.8)	2 (12.5)	1 (5.0)	2 (14.3)	
Other	9 (11.7)	5 (18.5)	3 (18.8)	0 (0.0)	1 (7.1)	
Postoperative treatment						0.039
None	21 (27.3)	8 (29.6)	3 (18.8)	4 (20.0)	6 (42.9)	
Chemotherapy	24 (31.2)	12 (44.4)	5 (31.3)	3 (15.0)	4 (28.6)	
Radiation therapy	15 (19.5)	6 (22.2)	1 (6.3)	6 (30.0)	2 (14.3)	
Chemo-radiation therapy	17 (22.1)	1 (3.7)	7 (43.8)	7 (35.0)	2 (14.3)	

PD: poorly differentiated carcinoma, AD: adenocarcinoma, SQ: squamous cell carcinoma, UD: undifferentiated carcinoma.

Bold represents p<0.05.

### Expression of metabolism-related proteins in PUMC according to the histologic and clinical subtype

**Figure 1:**
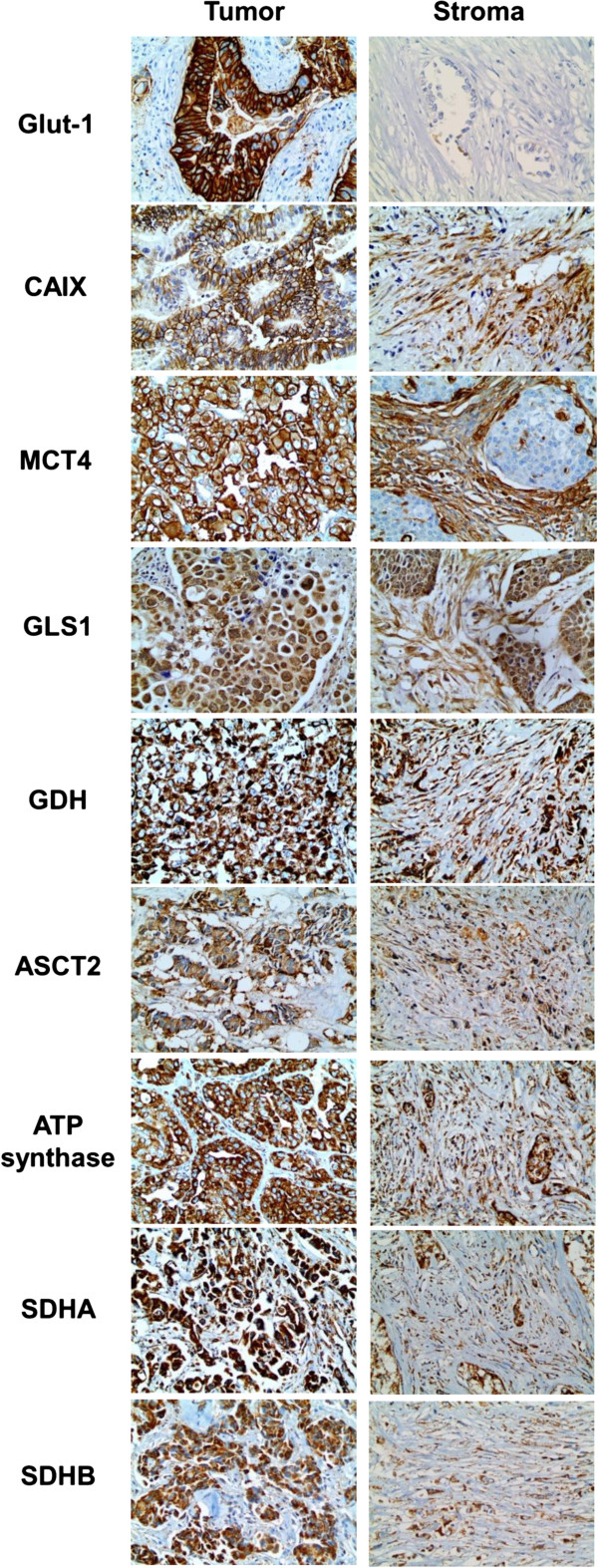
Expression of metabolism-related proteins in tumor and stroma of PUMC.

**Figure 2:**
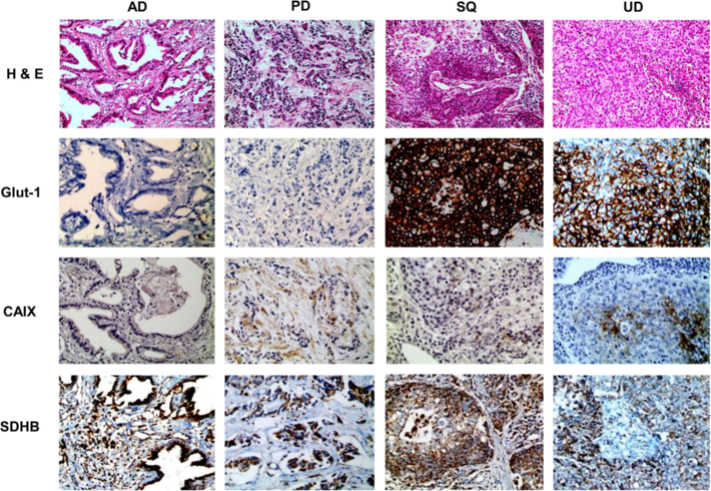
**Glut-1 and SDHB expression according to the histologic subtype of PUMC.** Compared to other subtypes, squamous cell carcinomas (SQ) expressed more Glut-1 (p = 0.028), while adenocarcinomas (AD) expressed more SDHB in the stroma. PD: poorly differentiated carcinoma, AD: adenocarcinoma, SQ: squamous cell carcinoma, UD: undifferentiated carcinoma.

Immunohistochemical staining on metabolism-related proteins except on Glut-1 in PUMC showed prominent expression on tumor and stroma (Figure 1), which didn’t show expression on stromal compartment. Table 2 and Figure 2 show the expression of metabolism-related proteins according to histologic subtypes. Compared to other subtypes, SQ expressed more Glut-1 (p = 0.028), while AD expressed more succinate dehydrogenase (SDH)B in its stroma (p = 0.025). Also, PD expressed more carbonic anhydrase (CA)IX in stroma compared to other subtypes (p = 0.050).

**Table 2:** Expression of metabolism-related proteins in PUMC according to histologic subtypes

**Immunohistochemical parameters**	**Total N = 77 (%)**	**Histologic subtype**				**p-value**
		**AD (n = 27) (%)**	**PD (n = 16) (%)**	**SQ (n = 20) (%)**	**UD (n = 14) (%)**	
Glut-1 (T)						**0.028**
Negative	41 (53.2)	17 (63.0)	11 (68.8)	5 (25.0)	8 (57.1)	
Positive	36 (46.8)	10 (37.0)	5 (31.3)	15 (75.0)	6 (42.9)	
Glut-1 (S)						N/A
Negative	77 (100.0)	27 (100.0)	16 (100.0)	20 (100.0)	14 (100.0)	
Positive	0 (0.0)	0 (0.0)	0 (0.0)	0 (0.0)	0 (0.0)	
CAIX (T)						0.124
Negative	68 (88.3)	24 (88.9)	15 (93.8)	15 (75.0)	14 (100.0)	
Positive	9 (11.7)	3 (11.1)	1 (6.3)	5 (25.0)	0 (0.0)	
CAIX (S)						**0.050**
Negative	75 (97.4)	27 (100.0)	14 (87.5)	20 (100.0)	14 (100.0)	
Positive	2 (2.6)	0 (0.0)	2 (12.5)	0 (0.0)	0 (0.0)	
MCT4 (T)						0.409
Negative	22 (28.6)	8 (29.6)	7 (43.8)	4 (20.0)	3 (21.4)	
Positive	55 (71.4)	19 (70.4)	9 (56.3)	16 (80.0)	11 (78.6)	
MCT4 (S)						0.902
Negative	43 (55.8)	15 (55.6)	10 (62.5)	10 (50.0)	8 (57.1)	
Positive	34 (44.2)	12 (44.4)	6 (37.5)	10 (50.0)	6 (42.9)	
GLS1 (T)						0.184
Negative	46 (59.7)	14 (51.9)	11 (68.8)	15 (75.0)	6 (42.9)	
Positive	31 (40.3)	13 (48.1)	5 (31.3)	5 (25.0)	8 (57.1)	
GLS1 (S)						0.401
Negative	68 (88.3)	23 (85.2)	13 (81.3)	18 (90.0)	14 (100.0)	
Positive	9 (11.7)	4 (14.8)	3 (18.8)	2 (10.0)	0 (0.0)	
GDH (T)						0.510
Negative	8 (10.4)	2 (7.4)	3 (18.8)	1 (5.0)	2 (14.3)	
Positive	69 (89.6)	25 (92.6)	13 (81.3)	19 (95.0)	12 (85.7)	
GDH (S)						0.350
Negative	58 (75.3)	18 (66.7)	11 (68.8)	17 (85.0)	12 (85.7)	
Positive	19 (24.7)	9 (33.3)	5 (31.3)	3 (15.0)	2 (14.3)	
ASCT2 (T)						0.413
Negative	47 (61.0)	15 (55.6)	8 (50.0)	15 (75.0)	9 (64.3)	
Positive	30 (39.0)	12 (44.4)	8 (50.0)	5 (25.0)	5 (35.7)	
ASCT2 (S)						0.313
Negative	73 (94.8)	24 (88.9)	16 (100.0)	19 (95.0)	14 (100.0)	
Positive	4 (5.2)	3 (11.1)	0 (0.0)	1 (5.0)	0 (0.0)	
ATP synthase (T)						0.597
Negative	2 (2.6)	1 (3.7)	1 (6.3)	0 (0.0)	0 (0.0)	
Positive	75 (97.4)	26 (96.3)	15 (93.8)	20 (100.0)	14 (100.0)	
ATP synthase (S)						0.101
Negative	62 (80.5)	18 (66.7)	13 (81.3)	19 (95.0)	12 (85.7)	
Positive	15 (19.5)	9 (33.3)	3 (18.8)	1 (5.0)	2 (14.3)	
SDHA (T)						0.208
Negative	3 (3.9)	1 (3.7)	2 (12.5)	0 (0.0)	0 (0.0)	
Positive	74 (96.1)	26 (96.3)	14 (87.5)	20 (100.0)	14 (100.0)	
SDHA (S)						0.200
Negative	59 (76.6)	17 (63.0)	14 (87.5)	16 (80.0)	12 (85.7)	
Positive	18 (23.4)	10 (37.0)	2 (12.5)	4 (20.0)	2 (14.3)	
SDHB (T)						0.112
Negative	28 (36.4)	7 (25.9)	9 (56.3)	9 (45.0)	3 (21.4)	
Positive	49 (63.6)	20 (74.1)	7 (43.8)	11 (55.0)	11 (78.6)	
SDHB (S)						**0.025**
Negative	66 (85.7)	19 (70.4)	15 (93.8)	20 (100.0)	12 (85.7)	
Positive	11 (14.3)	8 (29.6)	1 (6.3)	0 (0.0)	2 (14.3)	

PD: poorly differentiated carcinoma, AD: adenocarcinoma, SQ: squamous cell carcinoma, UD: undifferentiated carcinoma.

Bold represents p<0.05.

Table 3 shows the expression of metabolism-related proteins depending on clinical subtypes. The expression of Glut-1 in tumor was different for each of the clinical types: the nodal type expressed Glut-1 at the highest level, and the carcinomatosis type expressed Glut-1 at the lowest level (p = 0.021).

**Table 3:** Expression of metabolism-related proteins in PUMC according to clinical subtypes

**Immunohistochemical parameters**	**Total (n = 77) (%)**	**Clinical subtype**				**p-value**
		**Nodal type (n = 34) (%)**	**Single organ type (n = 15) (%)**	**Intermediate type (n = 14) (%)**	**Carcinomatosis type (n = 14)(%)**	
Glut-1 (T)						**0.021**
Negative	41 (53.2)	13 (38.2)	9 (60.0)	9 (64.3)	10 (71.4)	
Positive	36 (46.8)	21 (61.8)	6 (40.0)	5 (35.7)	4 (28.6)	
Glut-1 (S)						N/A
Negative	77 (100.0)	34 (100.0)	15 (100.0)	14 (100.0)	14 (100.0)	
Positive	0 (0.0)	0 (0.0)	0 (0.0)	0 (0.0)	0 (0.0)	
CAIX (T)						0.372
Negative	68 (88.3)	28 (82.4)	15 (100.0)	12 (85.7)	13 (92.9)	
Positive	9 (11.7)	6 (17.6)	0 (0.0)	2 (14.3)	1 (7.1)	
CAIX (S)						0.458
Negative	75 (97.4)	33 (97.1)	14 (93.3)	14 (100.0)	14 (100.0)	
Positive	2 (2.6)	1 (2.9)	1 (6.7)	0 (0.0)	0 (0.0)	
MCT4 (T)						0.877
Negative	22 (28.6)	11 (32.4)	2 (13.3)	4 (28.6)	5 (35.7)	
Positive	55 (71.4)	23 (67.6)	13 (86.7)	10 (71.4)	9 (64.3)	
MCT4 (S)						0.372
Negative	43 (55.8)	17 (50.0)	9 (60.0)	8 (57.1)	9 (64.3)	
Positive	34 (44.2)	17 (50.0)	6 (40.0)	6 (42.9)	5 (35.7)	
GLS1 (T)						0.176
Negative	46 (59.7)	23 (67.6)	8 (53.3)	9 (64.3)	6 (42.9)	
Positive	31 (40.3)	11 (32.4)	7 (46.7)	5 (35.7)	8 (57.1)	
GLS1 (S)						0.123
Negative	68 (88.3)	31 (91.2)	14 (93.3)	13 (92.9)	10 (71.4)	
Positive	9 (11.7)	3 (8.8)	1 (6.7)	1 (7.1)	4 (28.6)	
GDH (T)						0.181
Negative	8 (10.4)	2 (5.9)	2 (13.3)	1 (7.1)	3 (21.4)	
Positive	69 (89.6)	32 (94.1)	13 (86.7)	13 (92.9)	11 (78.6)	
GDH (S)						0.816
Negative	58 (75.3)	27 (79.4)	10 (66.7)	10 (71.4)	11 (78.6)	
Positive	19 (24.7)	7 (20.6)	5 (33.3)	4 (28.6)	3 (21.4)	
ASCT2 (T)						0.823
Negative	47 (61.0)	19 (55.9)	11 (73.3)	9 (64.3)	8 (57.1)	
Positive	30 (39.0)	15 (44.1)	4 (26.7)	5 (35.7)	6 (42.9)	
ASCT2 (S)						0.255
Negative	73 (94.8)	33 (97.1)	15 (100.0)	12 (85.7)	13 (92.9)	
Positive	4 (5.2)	1 (2.9)	0 (0.0)	2 (14.3)	1 (7.1)	
ATP synthase (T)						0.626
Negative	2 (2.6)	1 (2.9)	0 (0.0)	0 (0.0)	1 (7.1)	
Positive	75 (97.4)	33 (97.1)	15 (100.0)	14 (100.0)	13 (92.9)	
ATP synthase (S)						0.395
Negative	62 (80.5)	29 (85.3)	11 (73.3)	12 (85.7)	10 (71.4)	
Positive	15 (19.5)	5 (14.7)	4 (26.7)	2 (14.3)	4 (28.6)	
SDHA (T)						0.393
Negative	3 (3.9)	1 (2.9)	0 (0.0)	1 (7.1)	1 (7.1)	
Positive	74 (96.1)	33 (97.1)	15 (100.0)	13 (92.9)	13 (92.9)	
SDHA (S)						0.794
Negative	59 (76.6)	27 (79.4)	11 (73.3)	10 (71.4)	11 (78.6)	
Positive	18 (23.4)	7 (20.6)	4 (26.7)	4 (28.6)	3 (21.4)	
SDHB (T)						0.230
Negative	28 (36.4)	16 (47.1)	3 (20.0)	5 (35.7)	4 (28.6)	
Positive	49 (63.6)	18 (52.9)	12 (80.0)	9 (64.3)	10 (71.4)	
SDHB (S)						0.968
Negative	66 (85.7)	30 (88.2)	11 (73.3)	13 (92.9)	12 (85.7)	
Positive	11 (14.3)	4 (11.8)	4 (26.7)	1 (7.1)	2 (14.3)	

Bold represents p<0.05.

### Correlation between metabolic phenotypes of tumor clinicopathologic factors

When analyzing the clinicopathologic parameters based on the metabolic phenotypes based on the IHC results (Table 4), the involved organ of the PUMC was different for each mitochondrial type status. If the tumor showed a mitochondrial type, the tumor was most likely to be located at the lymph nodes (p = 0.026).

**Table 4:** Correlation between metabolic phenotypes of tumor and clinicopathologic factors

**Parameters**	**Glycolysis type**			**Glutaminolysis type**			**Mitochondrial type**		
	**Yes**	**No**	**p-value**	**Yes**	**No**	**p-value**	**Yes**	**No**	**p-value**
	**n = 33 (%)**	**n = 44 (%)**		**n = 43 (%)**	**n = 34 (%)**		**n = 74 (%)**	**n = 3 (%)**	
Age			0.087			0.340			1.000
<50	7 (21.2)	18 (40.9)		16 (37.2)	9 (26.5)		24 (32.4)	1 (33.3)	
≥50	26 (78.8)	26 (59.1)		27 (62.8)	25 (73.5)		50 (67.6)	2 (66.7)	
Sex			0.342			1.000			1.000
Male	23 (69.7)	25 (56.8)		27 (62.8)	21 (61.8)		46 (62.2)	2 (66.7)	
Female	10 (30.3)	19 (43.2)		16 (37.2)	13 (38.2)		28 (37.8)	1 (33.3)	
Clinical subtype			0.371			0.971			0.687
Nodal type	18 (54.5)	16 (36.4)		18 (41.9)	16 (47.1)		33 (44.6)	1 (33.3)	
Single organ type	5 (15.2)	10 (22.7)		9 (20.9)	6 (17.6)		15 (20.3)	0 (0.0)	
Intermediate type	6 (18.2)	8 (18.2)		8 (18.6)	6 (17.6)		13 (17.6)	1 (33.3)	
Carcinomatosis type	4 (12.1)	10 (22.7)		8 (18.6)	6 (17.6)		13 (17.6)	1 (33.3)	
Histologic type			0.093			0.545			0.208
AD	11 (33.3)	16 (36.4)		17 (39.5)	10 (29.4)		26 (35.1)	1 (33.3)	
PD	4 (12.1)	12 (27.3)		8 (18.6)	8 (23.5)		14 (18.9)	2 (66.7)	
SQ	13 (39.4)	7 (15.9)		9 (20.9)	11 (32.4)		20 (27.0)	0 (0.0)	
UP	5 (15.2)	9 (20.5)		9 (20.9)	5 (14.7)		14 (18.9)	0 (0.0)	
Organs involved			0.392			0.730			**0.026**
Lymph node	24 (72.7)	25 (56.8)		27 (62.8)	22 (64.7)		48 (64.9)	1 (33.3)	
Bone	3 (9.1)	7 (15.9)		7 (16.3)	3 (8.8)		10 (13.5)	0 (0.0)	
Brain	4 (12.1)	5 (11.4)		4 (9.3)	5 (14.7)		9 (12.2)	0 (0.0)	
Other	2 (6.1)	7 (15.9)		5 (11.6)	4 (11.8)		7 (9.5)	2 (66.7)	

Bold represents p<0.05.

When analyzing the clinicopathologic parameters according to the metabolic phenotypes of the stroma (Table 5), the involved organ depended on the stromal glutaminolysis status; when the stroma showed no glutaminolysis, the tumor most likely had invaded lymph node, bone, and brain, while the tumor was most likely to have invaded areas other than lymph node, bone, and brain when the stroma showed glutaminolysis (p = 0.003).

**Table 5:** Correlation between metabolic phenotypes of stroma and clinicopathologic factors

**Parameters**	**Glycolysis type**			**Glutaminolysis type**			**Mitochondrial type**		
	**Yes**	**No**	**p-value**	**Yes**	**No**	**p-value**	**Yes**	**No**	**p-value**
	**n = 2 (%)**	**n = 75 (%)**		**n = 8 (%)**	**n = 69 (%)**		**n = 74 (%)**	**n = 3 (%)**	
Age			0.547			0.710			0.741
<50	1 (50.0)	24 (32.0)		3 (37.5)	22 (31.9)		3 (25.0)	22 (33.8)	
≥50	1 (50.0)	51 (68.0)		5 (62.5)	47 (68.1)		9 (75.0)	43 (66.2)	
Sex			0.524			0.466			0.351
Male	2 (100.0)	46 (61.3)		4 (50.0)	44 (63.8)		6 (50.0)	42 (64.6)	
Female	0 (0.0)	29 (38.7)		4 (50.0)	25 (36.2)		6 (50.0)	23 (35.4)	
Clinical subtype			0.627			0.857			0.533
Nodal type	1 (50.0)	33 (44.0)		3 (37.5)	31 (44.9)		5 (41.7)	29 (44.6)	
Single organ type	1 (50.0)	14 (18.7)		1 (12.5)	14 (20.3)		4 (33.3)	11 (16.7)	
Intermediate type	0 (0.0)	14 (18.7)		2 (25.0)	12 (17.4)		1 (8.3)	13 (20.0)	
Carcinomatosis type	0 (0.0)	14 (18.7)		2 (25.0)	12 (17.4)		2 (16.7)	12 (18.5)	
Histologic type			**0.050**			0.237			**0.049**
AD	0 (0.0)	27 (36.0)		5 (62.5)	22 (31.9)		8 (66.7)	19 (29.2)	
PD	2 (100.0)	14 (18.7)		2 (25.0)	14 (20.3)		2 (16.7)	14 (21.5)	
SQ	0 (0.0)	20 (26.7)		1 (12.5)	19 (27.5)		0 (0.0)	20 (30.8)	
UP	0 (0.0)	14 (18.7)		0 (0.0)	14 (20.3)		2 (16.7)	12 (18.5)	
Organs involved			0.439			**0.003**			0.121
Lymph node	1 (50.0)	48 (64.0)		4 (50.0)	45 (65.2)		6 (50.0)	43 (66.2)	
Bone	1 (50.0)	9 (12.0)		0 (0.0)	10 (14.5)		3 (25.0)	7 (10.8)	
Brain	0 (0.0)	9 (12.0)		0 (0.0)	9 (13.0)		0 (0.0)	9 (13.8)	
Other	0 (0.0)	9 (12.0)		4 (50.0)	5 (7.2)		3 (25.0)	6 (9.2)	

Bold represents p<0.05.

Furthermore, the histologic subtypes were also different based on the stroma mitochondrial status. When the stroma was the mitochondrial type, the histologic subtype was mainly AD, but the non-mitochondrial type was associated more with SQ (p = 0.049). The cases with glycolysis-type stroma were all PD, while the cases with non-glycolysis stroma consisted of AD more often than others (p = 0.050).

The significant correlations between metabolic phenotype status were those between glutaminolysis type (T) and mitochondrial type (T) (r = 0.226, p = 0.048); and glutaminolysis type (S) and mitochondrial type (S) (r = 0.558, p = <0.001, Table 6).

**Table 6:** Correlation among metabolic phenotypes in tumor and stroma

	**Glycolysis type (T)**	**Glutaminolysis type (T)**	**Mitochondrial type (T)**	**Glycolysis type (S)**	**Glutaminolysis type (S)**	**Mitochondrial type (S)**
Glycolysis type (T)						
Correlation coefficient		-0.075	0.175	-0.141	-0.037	-0.083
p-value		0.514	0.129	0.220	0.750	0.475
Glutaminolysis type (T)						
Correlation coefficient			0.226	0.145	0.046	0.094
p-value			**0.048**	0.208	0.693	0.418
Mitochondrial type (T)						
Correlation coefficient				0.033	-0.151	0.087
p-value				0.777	0.189	0.454
Glycolysis type (S)						
Correlation coefficient					0.212	0.155
p-value					0.064	0.178
Glutaminolysis type (S)						
Correlation coefficient						0.558
p-value						**<0.001**

T, tumor, S, stroma.

Bold represents p<0.05.

In analysis of correlation between metabolism-related protein expression in tumor and stroma, there was a significant correlation in SDHB (T) and SDHB (S) (r = 0.309, p = 0.006), SDHB (T) and SDHA (S) (r = 0.290, p = 0.011), and SDHB (T) and ATP synthase (S) (r = 0.235, p = 0.039).

### Correlation between the expression of glycolysis related proteins and that of mitochondrial metabolism related proteins according to the histologic and clinical subtypes

On analysis of glycolysis related protein and mitochondrial metabolism related protein expression among histologic subtype, there was a significant correlation on AD in SDHB (S) and MCT4 (S) (r = 0.562, p = 0.002), SDHA (S) and MCT4 (S) (r = 0.549, p = 0.003), and MCT4 (S) and ATP synthase (S) (r = 0.632, p < 0.001), PD in SDHB (S) and CAIX (S) (r = 0.684, p = 0.004), SQ in SDHA (S) and MCT4 (S) (r = 0.500, p = 0.025), and UD in MCT4 (T) and SDHB (T) (r = 0.576, p = 0.031).

On analysis of expression of glycolysis related protein and mitochondrial metabolism in clinical subtype, there was a significant correlation in nodal type in SDHB (S) and CAIX (S) (r = 0.477, p = 0.004), SDHB (S) and MCT4 (S) (r = 0.365, p = 0.034), SDHA (S) and CAIX (S) (r = 0.342, p = 0.048), and SDHA (S) and MCT4 (S) (r = 0.364, p = 0.034), in single organ type in SDHB (S) and MCT4 (S) (r = 0.739, p = 0.002), SDHA (S) and MCT4 (S) (r = 0.739, p = 0.002), CAIX (S) and SDHB (T) (r = -0.535, p = 0.040), and MCT4 (S) and ATP synthase (S) (r = 0.739, p = 0.002), in carcinomatosis type in SDHB (S) and MCT4 (S) (r = 0.548, p = 0.043), SDHB (S) and MCT4 (T) (r = -0.548, p = 0.043), and MCT4 (S) and ATP synthase (S) (r = 0.849, p < 0.001), which was absent in intermediate type.

### Impact of clinicopathologic and immunohistochemical factors on prognosis

Analyzing the influence of clinicopathologic factors and the expression of metabolism-related proteins on the prognosis with univariate analysis, the factors associated with shorter overall survival were histologic subtype (UD > SQ > PD > AD, p = 0.002) and clinical type (single organ type > nodal type > intermediate type > carcinomatosis type, p < 0.001, Table 7), but the expression of metabolism-related proteins was not significantly related to the prognosis (Tables 8, 9).

**Table 7:** The impact of clinicopathologic parameters on prognosis by univariate analysis

**Clinicopathologic parameters**	No. of patients (n = 59^*^) (%)		**Overall survival**	
	**No. of cases**	**Patient death**	**Median survival (95% CI) (months)**	**P -value**
Age				0.326
<50	20	5	96 (70–123)	
≥50	39	13	43 (30–56)	
Sex				0.539
Male	35	10	88 (65–110)	
Female	24	8	74 (44–103)	
Histologic subtype				**0.002**
AD	21	11	23 (10–36)	
PD	13	1	34 (28–40)	
SQ	17	5	49 (33–65)	
UD	8	1	115 (86–145)	
Clinical subtype				**<0.001**
Nodal type	25	3	107 (88–126)	
Single organ type	10	1	119 (95–143)	
Intermediate type	10	4	21 (9–32)	
Carcinomatosis type	14	10	6 (3–10)	
Organs involved				0.701
Lymph node	38	12	77 (55–99)	
Bone	6	2	40 (18–62)	
Brain	7	1	31 (23–39)	
Other	8	3	83 (40–126)	
Postoperative treatment				0.638
None	7	1	107 (75–139)	
Chemotherapy	20	7	77 (45–108)	
Radiation therapy	15	4	52 (36–68)	
Chemo-radiation therapy	17	6	25 (18–33)	

PD: poorly differentiated carcinoma, AD: adenocarcinoma, SQ: squamous cell carcinoma, UD: undifferentiated carcinoma.

^*^Out of 77 patients, clinical follow-up data were available in 59 patients.

Bold represents p<0.05.

**Table 8:** The impact of expression status of metabolism-related proteins on prognosis by univariate analysis

**Clinicopathologic parameters**	No. of patients (n = 59^*^) (%)			**Overall survival**	
	**No. of cases**	**Patient death**		**Median survival (95% CI) (months)**	**P -value**
Glut-1 (T)					0.101
Negative	34	13		74 (49–98)	
Positive	25	5		55 (42–68)	
Glut-1 (S)			N/A		N/A
Negative	59	18		84 (66–102)	
Positive	0	0		N/A	
CAIX (T)					0.272
Negative	51	17		82 (63–101)	
Positive	8	1		54 (30–79)	
CAIX (S)					N/A
Negative	57	18		N/A	
Positive	2	0		N/A	
MCT4 (T)					0.759
Negative	19	5		87 (59–115)	
Positive	40	13		80 (57–103)	
MCT4 (S)					0.631
Negative	34	9		94 (73–115)	
Positive	25	9		43 (29–57)	
GLS1 (T)					0.139
Negative	39	10		49 (38–60)	
Positive	20	8		73 (42–103)	
GLS1 (S)					0.721
Negative	50	15		84 (65–104)	
Positive	9	3		46 (22–69)	
GDH (T)					1.000
Negative	7	2		28 (14–39)	
Positive	52	16		85 (66–104)	
GDH (S)					0.786
Negative	44	13		90 (72–109)	
Positive	15	5		40 (19–60)	
ASCT2 (T)					0.506
Negative	38	13		78 (54–101)	
Positive	21	5		36 (27–45)	
ASCT2 (S)					0.227
Negative	56	16		87 (68–105)	
Positive	3	2		16 (1–32)	
ATP synthase (T)					0.653
Negative	2	1		10 (10–10)	
Positive	57	17		85 (67–104)	
ATP synthase (S)					0.535
Negative	48	14		88 (68–107)	
Positive	11	4		23 (12–34)	
SDHA (T)					0.906
Negative	3	1		28 (13–43)	
Positive	56	17		84 (66–103)	
SDHA (S)					0.588
Negative	44	13		90 (70–109)	
Positive	15	5		37 (15–58)	
SDHB (T)					0.166
Negative	23	5		86 (56–115)	
Positive	36	13		79 (57–102)	
SDHB (S)					0.923
Negative	51	16		84 (65–103)	
Positive	8	2		27 (16–37)	

^*^Out of 77 patients, clinical follow-up data were available in 59 patients.

**Table 9:** The impact of metabolic phenotypes on prognosis by univariate analysis

**Clinicopathologic parameters**	No. of patients (n = 59^*^) (%)		**Overall survival**	
	**No. of cases**	**Patient death**	**Median survival (95% CI) (months)**	**P -value**
Tumor metabolic type				0.279
Glycolysis type	22	5	52 (37–67)	
Non-glycolysis type	37	13	79 (56–102)	
Tumor metabolic type				0.644
Glutaminolysis type	30	10	84 (60–108)	
Non-glutaminolysis type	29	8	45 (30–59)	
Tumor metabolic type				0.906
Mitochondrial type	56	17	84 (66–103)	
Non-mitochondrial type	3	1	28 (13–43)	
Stroma metabolic type				n/a
Glycolysis type	2	0	n/a	
Non-glycolysis type	57	18	n/a	
Stroma metabolic type				0.363
Glutaminolysis type	7	1	62 (43–80)	
Non-glutaminolysis type	52	17	80 (60–100)	
Stroma metabolic type				0.864
Mitochondrial type	9	2	28 (18–37)	
Non-mitochondrial type	50	16	83 (64–103)	

^*^ Out of 77 patients, clinical follow-up data were available in 59 patients.

**Figure 3:**
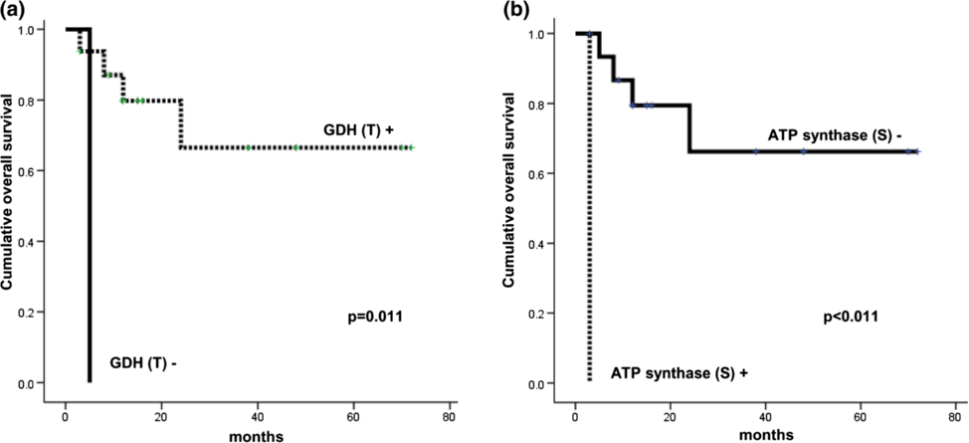
**Overall survival curves according to tumoral GDH (a) and stromal ATP synthase (b) status in the SQ group of PUMC.** T, tumor, S, stroma.

The influence of metabolism-related protein expression on the prognosis in each subgroup, categorized based on histologic subtype, was analyzed with univariate analysis. For SQ, tumoral glutamate dehydrogenase (GDH) negativity (p = 0.011) and stromal ATP synthase positivity (p < 0.001) were associated with shorter overall survival (Figure 3).

## Discussion

This study investigated for the metabolic features of PUMC in 77 cases. The expression of metabolism-related proteins was different for each of the histologic subtypes: tumoral Glut-1 expression was highest in SQ (p = 0.028). Because the metabolic features of PUMC have not been commonly reported in the literature, it is difficult to draw definitive conclusions. However, Glut-1, which is thought to function as a glucose transporter during glycolysis, is expressed the more vigorously in tumors with more aggressive tumor characteristics, such as high grade, high proliferative activity, and poor prognosis [10-17]. It can be deduced that SQ is likely the tumor with greatest glycolytic activity as it expresses Glut-1 the most among all the subtypes of PUMC. The expression of Glut-1 in SQ invading organs in cases other than PUMC is reported in cases of head and neck cancer [18,19] and esophageal cancer [13]. The expression of Glut-1 is associated with deep invasion, lymph node metastasis, and poor prognosis [13,18,19]. Glut-1 expression is also correlated to tumor aggressiveness in SQ.

In this study, stromal SDHB was expressed more in AD (p = 0.025). It is interesting that the degree of metabolism-related protein expression in the stroma near the tumor differed according to the histologic tumor subtypes. Some studies have clarified that there is an interaction between tumor and stroma with respect to tumor metabolism. The representative theory is the reverse-Warburg effect theory, which says that lactate created by glycolysis in the stroma is transferred to tumor cells and converted into energy through the TCA cycle by the mitochondria [20]. Consequently, PUMC can manifest an interaction between tumor and stroma. In this study, AD showed higher expression of mitochondrial markers, such as SDHD, in stroma, and the ratio of mitochondrial-type stroma, defined based on the expression of mitochondrial related proteins, was higher in AD than in SQ. Further study of the metabolic interaction between tumor and stroma in PUMC with different histologic subtypes will be critical.

In this study, the expression of Glut-1 in tumor varied for each of the clinical subtypes; the nodal type showed the highest expression, but the carcinomatosis type showed the least (p = 0.021). According to the literature, Glut-1 is strongly related to lymph node metastasis of tumors [21,22], so the higher expression in nodal type cases coincides with this data. Unusually, for the cases with extensive tumor involvement, including carcinomatosis type, the expression of Glut-1 was relatively low. One probable explanation is that the carcinomatosis type of PUMC may have discrete metabolic characteristics when compared to the carcinomatosis type of tumor with known primary cancer. When the primary carcinoma is known, the ratio of distant metastasis to more than 3 organs is less than 15%, but this ratio for PUMC is about 30% [23]. Moreover, the invaded organs in the PUMC cases are unusual locations, including kidney, adrenal gland, skin, and heart [24-26]. Thus, the carcinomatosis type of PUMC and primary carcinoma could be two very distinct disease entities with very different metabolic natures. Further study is required to substantiate this possibility. Another probable explanation is that the change in the metabolic characteristics for end-stage cancer. In previous studies, FDG-PET CT uptake clearly correlated to the expression of Glut-1 in tissue in early-stage cancer, while the correlation was poor for late-stage cancer [27]. While requiring further study, it can be speculated that metabolic characteristics change as a tumor progresses on to later stages.

In this study, tumoral GDH negativity and stromal ATP synthase positivity showed correlation to shorter OD. It is difficult to compare, since there is no previous study on PUMC and its metabolic characteristic. However, there are several studies reporting metabolism-related protein expression showing poor prognosis in various cancers [9-14]. Stromal GDH negativity was correlated to poor prognosis, especially in breast cancer [28], which showed compatible result with this study. In addition, ATP synthase expression in stroma showed relationship with poor prognosis, and the previous studies reported metabolism related protein and poor prognosis. Especially in breast cancer, the expression of glycolysis-related protein in stroma showed poor clinical outcome [20]. These stromal cells showing metabolic activities are defined as cancer-associated fibroblast, which show deprivement of cavelin-1 [20,29]. In PUMC, in contrast to breast cancer, ATP synthase expression, which is related to oxidative phosphorylation expression showed correlation to prognosis, which requires further study for nature of ATP synthase positive stromal cell.

The clinical significance of this study should be focused on two. First, its significance as a predictive factor. Even though small amount of biopsy tissue shows Glut-1 and/or SDHB expression in PUMC and predicts clinical, histologic subtype, prognosis by GDH, and/or ATP synthase expression in SQ type of PUMC, it needs further validation study. Secondly, its possibility as a treatment target in PUMC. Metabolism inhibitors, such as HIF-1α inhibitor [30,31], Glut-1 inhibitor [32,33], CAIX inhibitor [34], and monocarboxylate transporter (MCT)4 inhibitor [35], are reported in preclinical studies to suppress tumor growth in several tumor types. In this study, only the expression of metabolism-related protein on PUMC tissue is performed. Further validation study, including in vitro cell line study, mouse xenograft study, and clinical trial, is required to verify the possibility of metabolism inhibitor, especially Glut-1 inhibitor in target therapy of PUMC.

The main limitation of this study can be the small number of cases, and too various subtypes resulting in a small number for each classifications. Thus, there needs to be further study with case expansion. However, considering previous studies on PUMC studies with the case number of 26 ~ 100, the case number of this study does not necessarily have inferior standing [36-40].

## Conclusion

For PUMC, the expression of metabolism-related proteins such as Glut-1 and SDHB is likely different in tumor or stroma depending on the clinical and histologic subtype. SQ showed high expression for Glut-1 in tumor, AD for SDHB in stroma and PD for CAIX in stroma. Higher Glut-1 expression was noted in the nodal type and lower Glut-1 expression in carcinomatosis type.

## Abbreviations

PUMC: Primary unknown metastatic carcinoma; AD: Adenocarcinomas; PD: Poorly differentiated carcinomas; SQ: Squamous cell carcinomas; UD: Undifferentiated carcinomas; FFPE: Formalin-fixed, paraffin-embedded; H&E: Hematoxylin and eosin; TMA: Tissue microarray; IHC: Immunohistochemistry; CA: Carbonic anhydrase; MCT: Monocarboxylate transporter; GLS1: Glutaminase1; GDH: Glutamate dehydrogenase; ASCT2: Amino acid transporter-2; SDH: Succinate dehydrogenase.

## Competing interests

The authors declare that they have no competing interests.

## Authors’ contributions

HMK participated in the design of the study and performed the statistical analysis and drafted the manuscript. DHK carried out the immunochemistry. WHJ participated in its design. JSK conceived the study, and participated in its design and coordination and helped to draft the manuscript. All authors read and approved the final manuscript.

## Supplementary Material

Additional file 1: Table S1 Clone, dilution, and source of antibodies used. Click here for file
