# Supplementary material for: Metabolic phenotypes in primary unknown metastatic carcinoma
Source: J Transl Med. 2014 Jan 6;12:2. doi: 10.1186/1479-5876-12-2 (PMC3895852; doi:10.1186/1479-5876-12-2)
Supplement: Additional file 1: Table S1 — Clone, dilution, and source of antibodies used. [file 1479-5876-12-2-S1.doc]

**Supplementary Table 1. Clone, dilution, and source of antibodies used**

| Antibody | Clone | Dilution | Source |
| --- | --- | --- | --- |
| Glycolysis related | | | |
| Glut-1 | SPM498 | 1:200 | Abcam, Cambridge, UK |
| CAIX | Polyclonal | 1:100 | Abcam, Cambridge, UK |
| MCT4 | Polyclonal | 1:100 | Santa Cruz, CA, USA |
| Glutaminolysis related | | | |
| GLS1 | polyclonal | 1:50 | Abcam, Cambridge, UK |
| GDH | polyclonal | 1:100 | Abcam, Cambridge, UK |
| ASCT2 | polyclonal | 1:100 | Abcam, Cambridge, UK |
| Mitochondrial related | | | |
| ATP synthase | 15H4C4 | 1:100 | Abcam, Cambridge, UK |
| SDHA | 2E3GC12FB2AE2 | 1:100 | Abcam, Cambridge, UK |
| SDHB | 21A11AE7 | 1:100 | Abcam, Cambridge, UK |

CA, carbonic anhydrase, MCT, monocarboxylate transporter, GLS1, glutaminase1, GDH, glutamate dehydrogenase, ASCT2, amino acid transporter-2, SDH, succinate dehydrogenase
